# Piperacillin/Tazobactam as Cause of Acute Generalized Exanthematous Pustulosis

**DOI:** 10.1155/2019/3273987

**Published:** 2019-04-24

**Authors:** Aikaterini Kyriakou, Sofia-Chrysovalantou Zagalioti, Aikaterini Patsatsi, Nikiforos Galanis, Elizabeth Lazaridou

**Affiliations:** ^1^Second Department of Dermatology and Venereology, Medical School, Aristotle University of Thessaloniki, Greece; ^2^Papageorgiou Hospital, Thessaloniki, Greece; ^3^Department of Orthopedics, Medical School, Aristotle University of Thessaloniki, Greece

## Abstract

Acute generalized exanthematous pustulosis (AGEP) is a rare cutaneous adverse reaction mainly attributed to antibiotics. It is characterized by numerous, nonfollicular, sterile pustules, arising on an exanthematous and edematous base. It is a serious adverse reaction accompanied by fever and leukocytosis. Piperacillin/tazobactam is indicated for the treatment of patients with moderate to severe infections. Herein is reported a case of AGEP caused by piperacillin/tazobactam. A 78-year-old woman with metastatic breast cancer was presented to the emergency department reporting fever and groin pain. The laboratory analysis and more specifically urine cultivation showed a urinary tract infection by* E. coli* with sensitivity to piperacillin/tazobactam. She had no known allergies. She was started on intravenous piperacillin/tazobactam; she improved clinically on the second day, but on the fourth day of intravenous therapy, she developed extensive pustular rash on the folds and anterior proximal thighs, accompanied by fever and neutrophilia. Piperacillin/tazobactam administration was interrupted and she was given prednisolone for ten days. The patient improved clinically and her laboratory tests returned to normal after two weeks. AGEP is an uncommon side effect of piperacillin/tazobactam treatment and there are few cases reported.

## 1. Introduction

Acute generalized exanthematous pustulosis (AGEP) is a rare and severe cutaneous adverse reaction. Its incidence is estimated to be one to five cases per million people per year [1]. The vast majority of the cases reported in the literature are attributed to antibiotics, mainly penicillin and macrolide [2]. However, viral infection, UV radiation, and hypersensitivity reaction to mercury have also been reported as uncommon causes of AGEP [3]. It is presented with an abrupt onset of numerous, nonfollicular, sterile pustules, arising on an erythematous and edematous base [4–6]. The lesions are preferentially located either on the face or on the intertriginous areas; then they spread to the trunk and limbs within a few hours [3]. Latency time for antibiotics is generally short, while for other drugs it is longer [1], and rash is accompanied by fever and leukocytosis with neutrophilia [2, 5, 6]. Piperacillin/tazobactam is an antibiotic indicated for the treatment of patients with moderate to severe infections [7]. A case of a patient who developed this rare condition with the use of piperacillin/tazobactam is presented and discussed.

## 2. Case Presentation

A 78-year-old, Caucasian female patient with metastatic breast cancer under chemotherapy was presented to the emergency department reporting fever, groin pain, vomiting (over 10 times per day) and being unable to get up of the bed for the past four days after the last chemotherapy. The initial laboratory workup revealed increased serum creatinine level of 3.20 mg/dl (GFR=14.89 mL/min/1.73m2, baseline= 0.57-1.11). Moreover, the urine analysis showed increased pyocytes (>100), while the urine cultivation highlighted* E. coli* with sensitivity to piperacillin/tazobactam.

**Figure 1:**
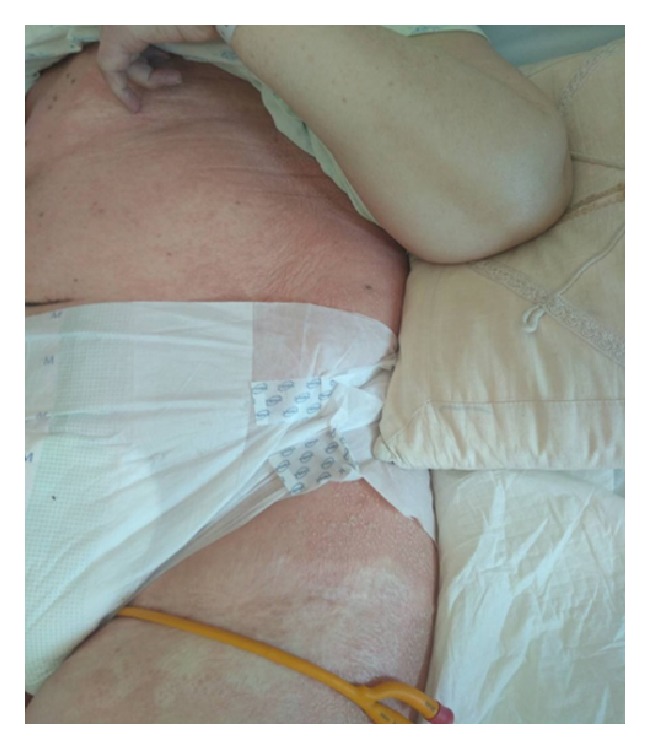
Extensive pustular rash located on the folds and proximal limp sessions.

**Figure 2:**
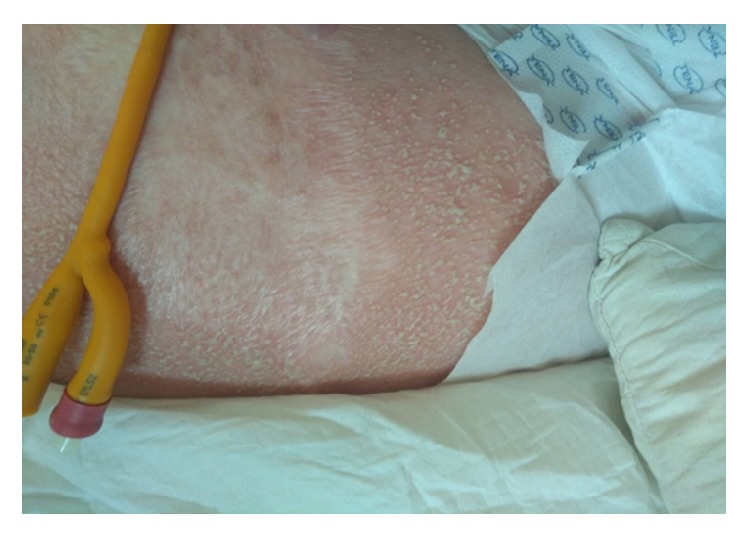
Numerous, pinhead-sized, nonfollicular, sterile pustules, arising on an erythematous and edematous base.

She was started on intravenous piperacillin/tazobactam (4.5g x 4, due to the impairment of renal function serum creatinine= 1.44 mg/dl) for urinary tract infection and at the same time she was kept hydrated. She had no known drug allergies and no history of psoriasis. She was clinically improved after two days of antibiotic therapy. On the fourth day of the intravenous piperacillin/tazobactam administration protocol, she abruptly developed extensive erythema and pustules that were located predominantly on the folds and anterior proximal thighs (Figures 1 and 2). The Nikolsky sign was negative and there was no mucosal involvement. The rash was accompanied by fever (38.5°C) and mild pruritus. The blood tests showed significant leukocytosis neutrophilia. Impressively, the white blood cells increased from 5.26 K/*μ*l (with neutrophils 3.5 K/*μ*l) to 39.6 x 10^3^ K/*μ*l (with neutrophils 27.7 K/*μ*l) within two days of developing the rash. The patient refused to undergo a skin biopsy. However, Tzanck smear showed mainly neutrophils accompanied by the presence of eosinophils and lymphocytes, without any bacterial cocci. Moreover, bacterial culture from pustule content was negative.

Based on the clinical and laboratory results, it was concluded that the patient presented AGEP. Moreover, based on the AGEP validation score of the EuroSCAR study group [1], our patient's score was 9, which is compatible with a definite diagnosis of AGEP. Piperacillin/tazobactam administration was interrupted (Day 4) and the patient was given 30 mg of prednisolone intravenously once a day, which was tapered and stopped within 10 days. Both the skin rash resolved, followed by postpustular desquamation, and the white blood cells returned to their normal levels two weeks after the discontinuation of the drug. Based on the WHO-UMC causality categories, association of the described side effect to the culprit drug could be characterized as “probable/likely.” The limitation of this work was the fact that there was no systemic rechallenge.

## 3. Discussion

AGEP was first described by Beylot et al. in 1980 [8], while in 1991 Roujeau et al. characterized the disease as a drug-induced, severe reaction and differentiated it from pustular psoriasis [3].

Until 2000, AGEP was characterized by the diagnostic triad nonfollicular, intraepidermal, or subcorneal pustules, fever greater than 38C, and neutrophil count above 7000 cells/mL [9]. Since 2001, the diagnosis of AGEP is based on morphology, course, and histology [1]. The mucous membranes are not frequently affected.

Overlap of AGEP and toxic epidermal necrolysis is a nonexisting diagnosis [10]. Cases of AGEP and drug-related rash with eosinophilia and systemic symptoms syndrome have previously been reported [11]. Biopsy should be performed, whenever is possible, since histopathology is part of the validation score of AGEP [1]. Moreover, there are histopathological, diagnostic clues in favor of AGEP in patients with a pustular eruption [12].

AGEP is suggested to be type IV hypersensitivity reaction which is mediated by T cells [13]. Recently, Pinho et al. reported that patch testing is a safe and useful tool for confirming the culprit drug involvement in most nonimmediate cutaneous adverse drug reactions from antibiotics [14]. Based on their findings, in AGEP cases, patch tests were mainly useful in detecting reactivity to fluoroquinolones. Moreover, a negative patch test result cannot be used to exclude drug imputability, since overall reactivity was relatively low [14]. In our case, no patch test was performed, which places a limitation.

The necessity of steroids in the treatment of AGEP is still a matter of debate [1]. However, except for cessation of the causative agent, close monitoring based on the immune status of the patient is indicated, since AGEP is not always easily controlled [12].

AGEP is a rarely reported severe cutaneous adverse drug reaction [11, 15]. In general, AGEP has a self-limited course lasting ≤ 15 days and its treatment lies mainly in withdrawing the offending drug. However, systematically administered corticosteroids may be necessary in refractory or severe cases.

## References

[1] Sidoroff A., Halevy S., Bavinck J. N. B., Vaillant L., Roujeau J.-C. (2001). Acute generalized exanthematous pustulosis (AGEP)—a clinical reaction pattern. *Journal of Cutaneous Pathology*.

[2] De A., Das S., Sarda A., Pal D., Biswas P. (2018). Acute generalised exanthematous pustulosis: An update. *Indian Journal of Dermatology*.

[3] Roujeau J.-C., Bioulac-Sage P., Bourseau C. (1991). Acute generalized exanthematous pustulosis: analysis of 63 cases. *JAMA Dermatology*.

[4] Thienvibul C., Vachiramon V., Chanprapaph K. (2015). Five-year retrospective review of acute generalized exanthematous pustulosis. *Dermatology Research and Practice*.

[5] Lin Y., Yang C., Sindy H. (2014). Severe cutaneous adverse reactions related to systemic antibiotics. *Clinical Infectious Diseases*.

[6] Choi M. J., Kim H. S., Park H. J. (2010). Clinicopathologic manifestations of 36 Korean patients with acute generalized exanthematous pustulosis: a case series and review of the literature. *Annals of Dermatology*.

[7] McCormick H., Tomaka N., Baggett S. (2015). Comparison of acute renal injury associated with intermittent and extended infusion piperacillin/tazobactam. *American journal of health-system pharmacy : AJHP : official journal of the American Society of Health-System Pharmacists*.

[8] Beylot C., Bioulac P., Doutre M. S. (1980). Acute generalized exanthematic pustuloses (four cases) (author's transl)]. *Ann Dermatol Venereol*.

[9] Roujeau J.-C. (2000). Neutrophilic drug eruptions. *Clinics in Dermatology*.

[10] Van Hattem S., Beerthuizen G. I., Kardaun S. H. (2014). Severe flucloxacillin-induced acute generalized exanthematous pustulosis (AGEP), with toxic epidermal necrolysis (TEN)-like features: Does overlap between AGEP and TEN exist? Clinical report and review of the literature. *British Journal of Dermatology*.

[11] Kim T. I., Jeong K. H., Shin M. K., Kim N. I. (2016). Piperacillin/tazobactam-associated hypersensitivity syndrome with overlapping features of acute generalized exanthematous pustulosis and drug-related rash with eosinophilia and systemic symptoms syndrome. *Annals of Dermatology*.

[12] Halevy S., Kardaun S. H., Davidovici B., Wechsler J. (2010). The spectrum of histopathological features in acute generalized exanthematous pustulosis: A study of 102 cases. *British Journal of Dermatology*.

[13] Pichler W. J. (2003). Delayed Drug Hypersensitivity Reactions. *Annals of Internal Medicine*.

[14] Pinho A., Coutinho I., Gameiro A., Gouveia M., Gonçalo M. (2017). Patch testing – a valuable tool for investigating non-immediate cutaneous adverse drug reactions to antibiotics. *Journal of the European Academy of Dermatology and Venereology*.

[15] Talati S., Lala M., Kapupara H., Thet Z. (2009). Acute generalized exanthematous pustulosis: A rare clinical entity with use of piperacillin/tazobactam. *American Journal of Therapeutics*.

